# White Cord Syndrome: Myelopathy Caused by an Ossified Posterior Longitudinal Ligament After Posterior Cervical Laminectomy and Fusion

**DOI:** 10.7759/cureus.80295

**Published:** 2025-03-09

**Authors:** Ashlie Maldonado-Pérez, Joyce Campos, Gisela Murray, Samuel Estronza, Emil A Pastrana

**Affiliations:** 1 Department of Neurosurgery, University of Puerto Rico, Medical Sciences Campus, San Juan, PRI; 2 Department of Neurosurgery, San Juan Bautista School of Medicine, Caguas, PRI

**Keywords:** injury, ischemia, neurospine, ossified posterior longitudinal ligament, reperfusion, spinal fusion, spinal stenosis, white cord syndrome

## Abstract

White cord syndrome (WCS) is a rare but serious postoperative complication after spinal decompression surgery. Risk factors such as advanced age, ossification of the posterior longitudinal ligament (OPLL), and major surgery may have predisposed this patient to develop WCS. This report discusses a 72-year-old Hispanic male patient with cervical stenosis due to OPLL who underwent posterior cervical decompression and fusion. After laminectomy and decompression were done, neuromonitoring signals decreased significantly. Despite intraoperative interventions after neuromonitoring signals decreased (e.g., mean arterial pressure augmentation with vasopressors, intravenous steroid therapy), the patient experienced significant quadriparesis following surgery. On postoperative imaging, spinal cord edema and T2 hyperintensities were noted on cervical MRI, consistent with the descriptions of WCS. The patient's postoperative course was marked by significant complications such as respiratory distress, hemodynamic instability, and infection, which ultimately led to his demise. This case highlights the necessity of individual risk factor stratification prior to surgery, careful neuromonitoring, and prompt treatment to manage suspected WCS.

## Introduction

The term “white cord syndrome” (WCS) was first mentioned by Chin et al. in 2013 to describe a suspected reperfusion injury in a patient after undergoing cervical decompression [1]. Reperfusion injuries result from rapid reoxygenation of ischemic tissue, causing oxidative stress, leukocyte infiltration, disruption of the blood-brain barrier, and complement activation, leading to tissue edema, neuronal death, and worsening neurological dysfunction; this mechanism has been proved in the brain and spine by different authors [2,3]. Although major neurological deficits are not common after spinal surgery, little is known about this etiology. Cramer et al. studied the complications after spinal surgery in 11,817 adults who underwent spinal surgeries from 1996 to 2006 and found that the overall incidence of neurological deficit after surgery was 0.178% [4]. Despite the low incidence of complications after spinal surgery, WCS causes serious and detrimental morbidities, and surgeons must be aware of its development. This report discusses the development and complications of WCS in the case of a 72-year-old Hispanic male patient after posterior cervical decompression and fusion. Other etiologies for the acute postoperative neurological deficit were discarded after thorough intraoperative evaluation of the surgical cavity by the surgeon and assistants revealed no significant findings. The surgery was uneventful, with no visible injury to the spinal cord. Additionally, postoperative imaging did not reveal signs of epidural hematoma, misplaced pedicle screws, inadequate decompression, or spinal cord arterial occlusion. Unfortunately, the patient expired due to complications of this condition. We will also describe the complications experienced with this patient.

## Case presentation

**Figure 1:**
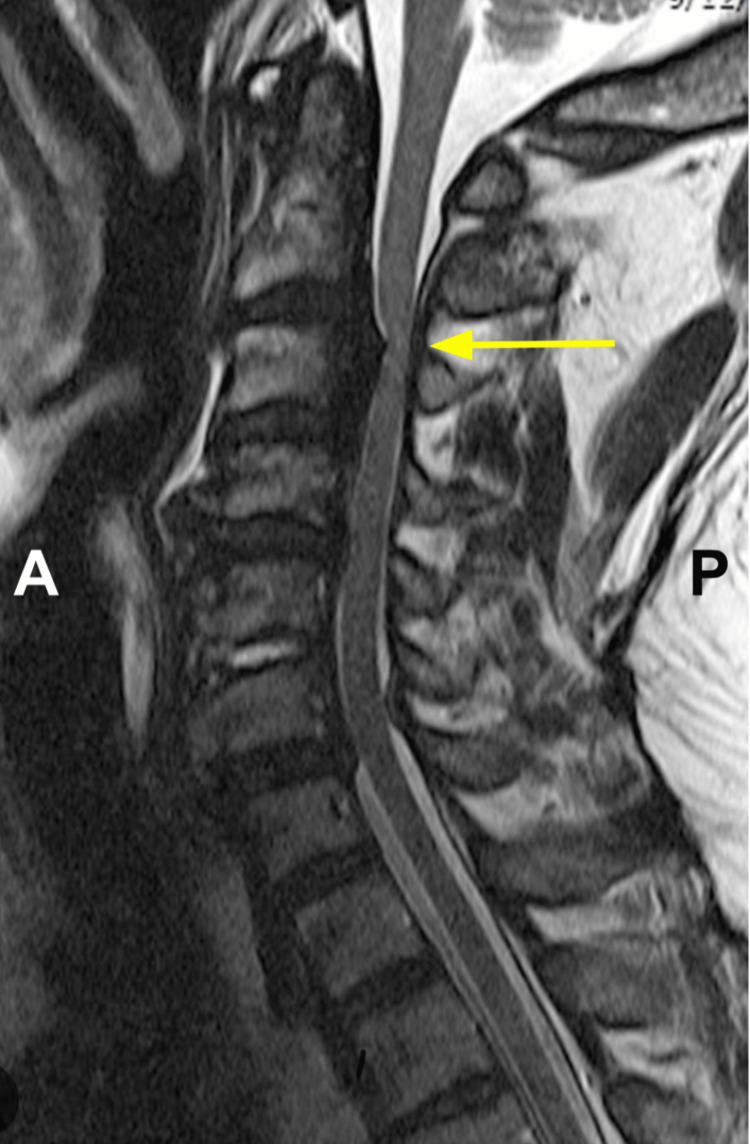
Sagittal view of preoperative cervical T2-weighted MRI. An ossified posterior longitudinal ligament is seen causing severe stenosis of the spinal canal at the level of C3-C4 vertebral bodies (yellow arrow). No T2-weighted hyperintensities noted in preoperative imaging. A: Anterior, P: Posterior

**Figure 2:**
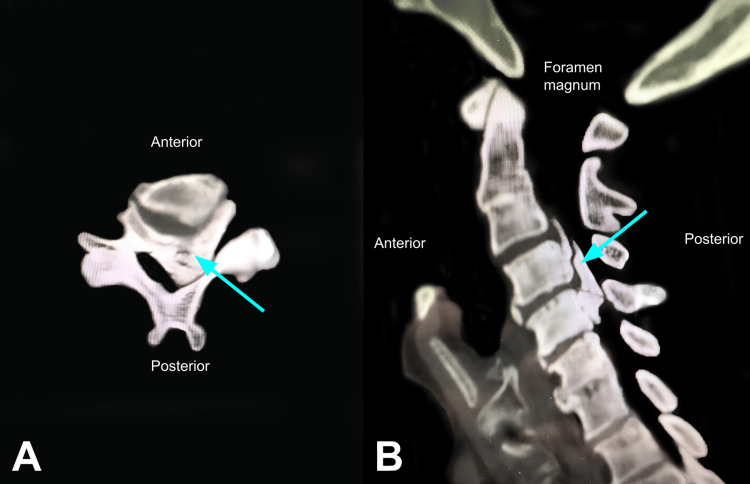
Axial (A) and sagittal (B) views of the cervical CT scan. Ossified posterior longitudinal ligament is seen causing severe stenosis of the spinal canal at the level of C3-C4 vertebral bodies (blue arrow)

A 72-year-old man with a past medical history of hypertension, dyslipidemia, diabetes mellitus, and a previous myocardial infarction presented to our clinic complaining of neck pain, imbalance, gait disturbances, bilateral 4/5 muscle strength grading lower and upper extremity weakness, and numbness. Cervical magnetic resonance imaging (MRI) and a computed tomography (CT) scan showed severe cervical stenosis, most notable at the level of C3-C4 vertebral bodies due to ossification of the posterior longitudinal ligament (OPLL) (Figures 1, 2A, 2B). He was scheduled for elective surgery for cervical decompression and instrumentation.

In the operating room, the patient was anesthetized and intubated, neuromonitoring devices were set up for real-time evaluation of the nervous system, and he was positioned prone with his head placed in a skull clamp with three-point fixation. There were no changes in somatosensory-evoked potentials (SSEPs), electromyography (EMG), or transcranial motor-evoked potentials (TcMEPs) after positioning. A midline incision was done with a 10-blade at the level of the vertebral bodies of C3 to C7, and a standard posterior cervical approach was performed. Dissection was done in anatomical layers, with complete exposure of C3-C7 posterior elements. Fluoroscopic imaging was used to confirm localization of the vertebral bodies. Using a free hand technique, screws were placed bilaterally at the C3, C4, C5, C6, and C7 lateral masses. The surgery is described as follows: (i) First, the pilot hole was drilled after correct anatomical localization with intraoperative fluoroscopy; (ii) The hole was expanded using a measured drill until it was of appropriate length; (iii) The hole was checked for medial, lateral, superior, inferior, and anterior breaches using a pedicle probe, with none identified; (IV) The C3-C4, C4-C5, C5-C6, and C6-C7 facet joints were decorticated along with lateral mass, and adequate length titanium rods were placed, secured, tightened, and torqued off; (V) Using a high-speed drill, C2/C3/C4/C5/C6/C7 laminectomies were done with facetectomy.

Throughout the surgery, the patient's TcMEPs were continuously monitored and updated, and the responses were compared to the post-induction baselines. The neurophysiologist visualized the upper and lower SSEP components, consisting of cortical, subcortical, and peripheral responses throughout the procedure. TcMEPs were elicited for all extremities prior to incision, with good responses globally.

Immediately after the laminectomies of C2, C3, C4, C5, C6, and C7 were done, cortical and subcortical neuromonitoring responses were reduced to 0/0 mA, TcMEPs and SSEPs were lost, and only peripheral responses were present. We started immediately with the spinal injury guidelines recommended by the American College of Surgeons [5]. Mean arterial pressure (MAP) was kept above 90 mmHg, and a loading dose of methylprednisolone of 30 mg/kg was administered over 15 minutes, followed by an infusion of methylprednisolone 5.4mg/kg/hr. This intervention with steroids was done because the surgeon suspected WCS. However, there was no improvement in neuromonitoring. Evaluation of the surgical cavity demonstrated no evidence of an epidural hematoma or physical compression by instrumentation or a fragment of bone. Emergency durotomy was done to decrease the intraspinal pressure and augment perfusion. The spinal cord was pulsating, and cerebrospinal fluid (CSF) egress was permitted judiciously. There was no evidence of visible cord contusion or discoloration. Durotomy was then closed in a watertight fashion with interrupted stitches. The surgical cavity was irrigated with three liters of 0.35% sterile iodine solution followed by three liters of 0.9% saline. Despite our efforts, TcMEPs were constantly tested with no improvement, even after wound closure. No other significant changes were seen in either the responses' latency or amplitude for the entire surgical procedure length (i.e., 330 minutes). Final intraoperative radiographs obtained demonstrated adequate placement of screws and rods. 

**Figure 3:**
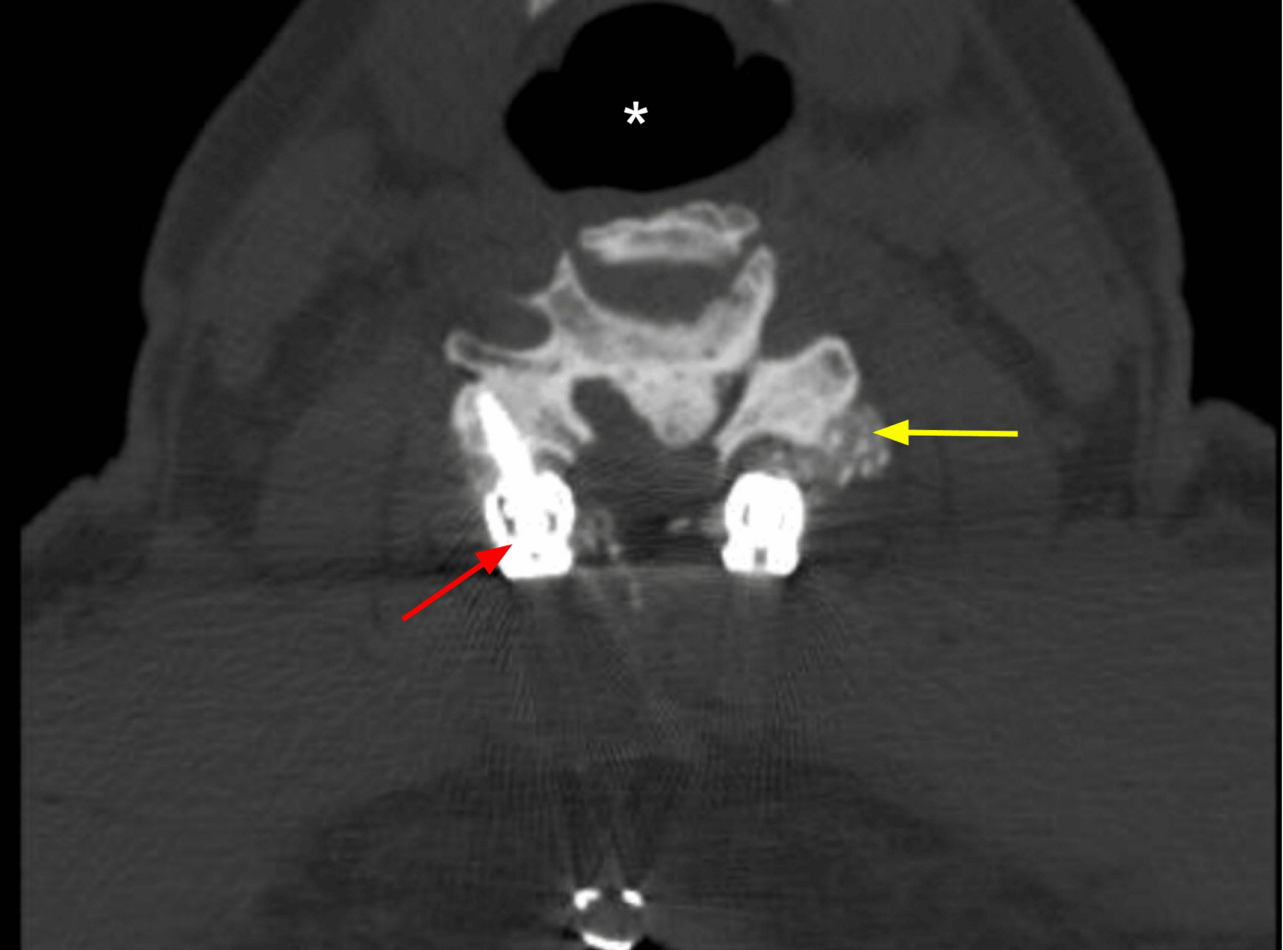
Axial view of the postoperative cervical CT scan. Imaging shows C4 vertebra with bone graft for fusion (yellow arrow) and correctly positioned screw in the lateral mass (red arrow). * trachea

**Figure 4:**
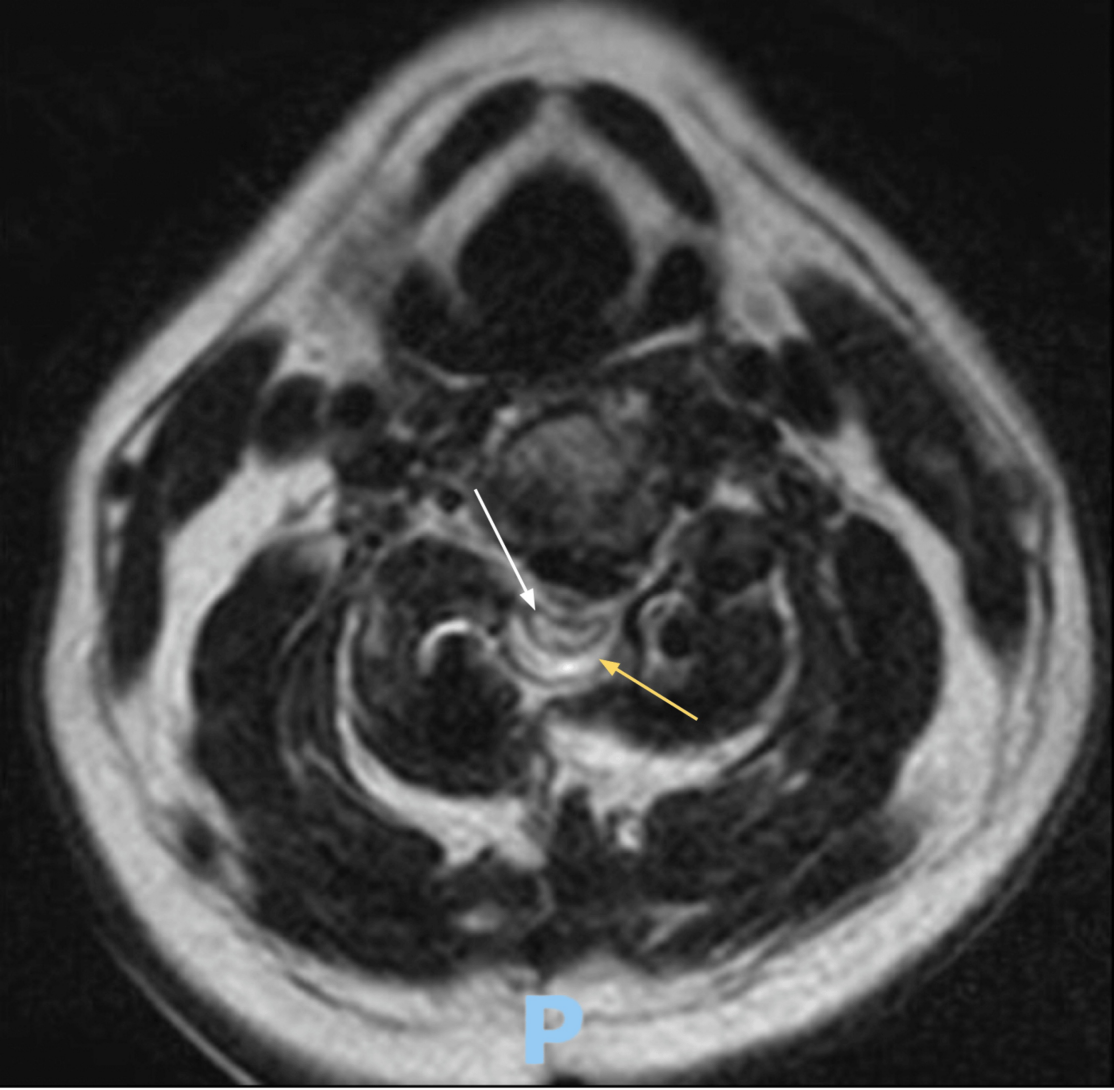
Axial view of the postoperative cervical T2-weighted MRI at the level of C3-C4. An adequate CSF signal around the spinal cord is seen, which suggests adequate decompression (yellow arrow). An intramedullary T2-weighted hyperintensity is noted in postoperative imaging (white arrow). P: Posterior

**Figure 5:**
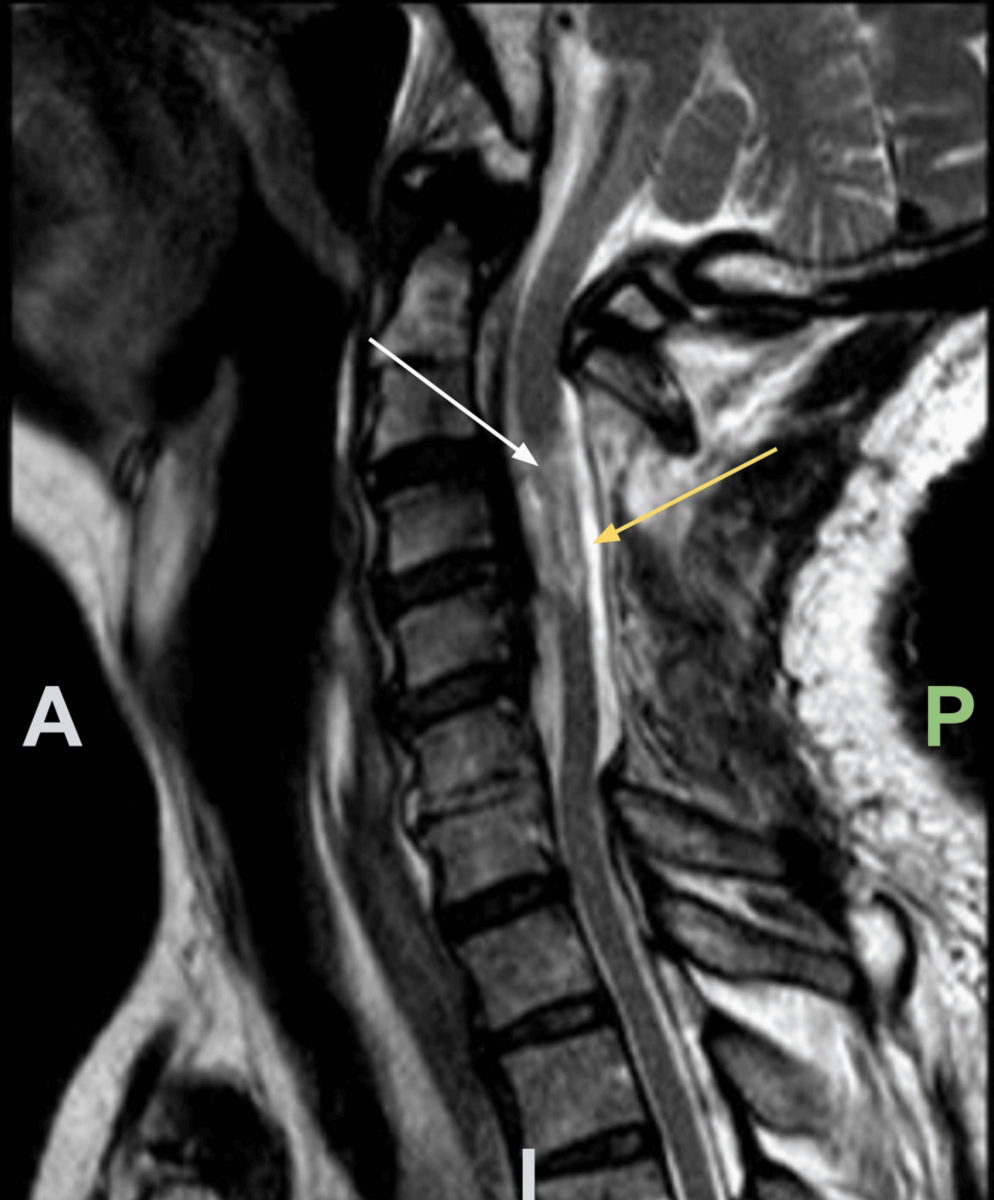
Sagittal view of postoperative cervical MRI, T2-weighted. An abnormal intramedullary T2 hyperintensity spanning from C3-C4 is seen (white arrow). CSF signal surrounding the spinal cord suggests adequate decompression (yellow arrow). A: Anterior, P: Posterior, I: Inferior

The patient was extubated successfully. On immediate postoperative physical examination (PE), the patient was found to have profound quadriparesis of 2/5 muscle strength grading in bilateral lower extremities (LEs) and 3/5 in left upper extremity (LUE); the right extremity was noted with plegia. An emergency postoperative cervical computed tomography (CT) scan and MRI were done. On the cervical CT scan, there was a cervical spine fusion with a bone graft of the C2-C4 vertebral bodies (Figure 3). Postoperative MRI is remarkable for an adequate amount of CSF signal surrounding the cervical cord at the surgical levels, suggesting adequate decompression (Figure 4). An abnormal intramedullary T2 hyperintensity spanning from C3-C4 was seen (Figures 4, 5). No syringohydromyelia was appreciated.

Postoperative course

The patient was transferred to the neurointensive care unit (NSICU) for continued management. On postoperative day (POD) 1, a PE revealed complete right upper extremity (RUE) plegia, LUE motor strength of 3/5, and bilateral LE motor strength of 2/5 in the distal muscle groups. The Glasgow Coma Scale (GCS) score was 15, and he reported no numbness or tingling. He remained on spinal cord injury treatment, receiving methylprednisolone at 5.4 mg/kg/hr for 48 hours postoperatively. The MAP target was maintained above 85 mmHg for seven consecutive days. The steroids were discontinued on POD 2. On POD 3, the patient developed sudden respiratory distress characterized by abdominal breathing and severe shortness of breath, necessitating emergent intubation. He also developed leukocytosis, with white blood cells of 23 thou/uL (range: 3.98-10.04 thou/uL). Blood and respiratory tract cultures were obtained. By POD 4, the patient remained on mechanical ventilation, with unchanged overall physical examination except for intermittent episodes of fever, prompting the initiation of empirical antibiotics. On POD 5, the patient experienced a neurological deterioration, with a decline in GCS from 11T to 8T (opening eyes to pain and localizing to pain), leading to an urgent head CT scan. However, his condition continued to worsen, and he became hemodynamically unstable, requiring aggressive titration of the norepinephrine drip and close monitoring in the NSICU. By the morning of POD 6, the patient’s respiratory distress worsened significantly, with oxygen desaturation of 81%-85% despite mechanical ventilation. A head CT and chest CT angiography were ordered to assess neurological deterioration and rule out pulmonary embolism, respectively. However, due to rapid deterioration and hemodynamic instability, the patient was unable to be transported for imaging. Later that day, the patient developed shock and continued to receive empirical antibiotic treatment. Despite increasing the fraction of inspired oxygen (FiO2) to 100% in the ventilator, the patient’s oxygenation continued to decline. He subsequently developed hypotension, bradycardia, low MAPs, hematemesis, and anuria. A comprehensive evaluation, including an echocardiogram, chest auscultation, and assessment of the urinary catheter and endotracheal tube, yielded no significant findings. The patient’s condition continued to deteriorate, culminating in cardiac arrest. Despite aggressive resuscitation efforts, including vasopressor support, multiple doses of epinephrine, and approximately 20 minutes of CPR, the patient passed away.

## Discussion

The postoperative onset of major neurological deficits after spinal surgery is low. A study done by Cramer et al. revealed an approximate overall incidence of postoperative major neurological deficits of 0.178% [4]. Etiologies reported by the authors included epidural hematoma (the most commonly seen in their series), inadequate decompression, presumed vascular compromise, graft or cage dislodgement, and presumed surgical trauma [4]. In their study, vascular compromise was suspected in four of the 21 patients who suffered major neurological deficits. MRI of one patient showed an increased signal of the thoracic spinal cord. However, imaging done in the other three patients did not reveal any significant changes.

By 2019, only five cases of WCS were reported in the literature, all after anterior cervical decompression and fusion. Chin et al. published the first case and used the term “white cord syndrome” for the first time in 2013 [1]. Wiginton et al. presented the first case of transient quadriplegia following posterior cervical decompression and reviewed three cases of WCS published at the moment [6]. They found that all three cases of WCS presented with preoperative hyperintense signals on T2-weighted MRI. In all three cases, postoperative MRI revealed a more pronounced T2-hyperintense signal. They treated the patients with increased MAP and dexamethasone, resulting in some improvement within 8-48 hours and eventual full recovery. In the case report by Wiginton et al., the patient experienced a progressive loss of somatosensory evoked potentials (SSEPs) in the hands shortly after the resection of the C1 vertebral arch, which subsequently extended to all extremities [6]. Loss of SSEPs was followed by the loss of MEPs. They stopped the surgery and raised the MAP > 95mmHg. Intraoperative fluoroscopy was negative for subluxation or malalignment. They performed C2 laminectomy as a precaution, after which SSEPs and MEPs started to return. He woke up from anesthesia with 1/5 in all four extremities and within minutes, he started recovering strength to up to 4/5. Postoperative MRI revealed an increase in the previously seen T2-weighted signal of the spinal cord. They continued treatment with MAP > 90mmHg, dexamethasone 10 mg intravenously (IV) every 6 hours, and rehabilitation in the intensive care unit (ICU). In all the cases studied by these authors, the T2-weighted hyperintense signal on spinal MRI was noted preoperatively.

Antwi et al. reported the second case of WCS in the literature after a posterior cervical decompression [7]. They hypothesize that the acute neurological deficit developed after decompression may be due to free radical oxygen species. The authors also state that direct trauma from the quick flow of blood back into the area may also have an effect on the development of WCS [7]. In their case, the patient had a postoperative nonspecific T2/FLAIR hyperintense intramedullary signal in MRI at the level of C5, more prominent in the left. Surgery went uneventfully until closure, when the neurophysiologist alerted the surgeon of the loss of MEPs in the left hand and leg. Despite the authors’ measures, the MEPs were permanently lost. After waking up from the anesthesia, the patient presented with left hemiplegia, 0/5 strength. The patient’s T2-weighted postoperative MRI was remarkable for increased T2-weighted/FLAIR hyperintense signal on the left side of the spinal cord, suggesting ischemia. They started the patient on methylprednisolone intravenously for 24 hours for the presumed diagnosis of WCS. By postoperative day 1, the patient already recovered strength to 3/5 in bilateral lower extremities and 4/5 in bilateral upper extremities. The authors state that improvement after high-dose steroids favors reperfusion injury, as steroids inhibit lipid peroxidation after spinal cord injury, as reported by Hall [8]. 

Busack and Eagleton reported the tenth case in the literature [9]. They presented a 63-year-old male patient with progressive difficulty walking, declining balance, and neuropathic pain of one-year progression. The patient underwent spinal surgery for severe cervical stenosis from C2-C3 to C5-C6, with an associated hyperintense intramedullary T2/FLAIR signal change in MRI. The surgery was uneventful. However, SSEPs were lost suddenly, and the spinal cord was noted to expand dramatically after decompression. WCS was suspected, for which the patient received dexamethasone 8 mg intravenously perioperatively. Postoperative physical examination was remarkable for lack of sensation below T3 and 0/5 in all muscle groups, except 4/5 in bilateral deltoid muscles. After multiple doses of steroids, he regained sensorium down to T10 level, and his strength increased significantly. It is known that CSF pressure increases after spinal cord decompression, which is proved intraoperatively by bulging of the thecal sac. One author's suggestion is to place a CSF drain preoperatively. This may aid in mitigating the elevated CSF pressure effects on spinal cord perfusion after spinal decompression [9]. 

In 2013, Karadimas et al. designed a rat model to mimic cervical spondylotic myelopathy. They identified that the microvessel network of the cervical spinal cord suffers changes under chronic compression, specifically loss of blood vessels and flattening of preserved vessels [10]. In 2015, the same author studied the role of riluzole, a sodium glutamate antagonist, in preventing ischemia-reperfusion injuries in rats undergoing spinal decompression surgery [11]. They found that spinal cord blood flow increased more than double after decompression surgery and that more neurons expressed 8oxoG DNA (a common by-product of DNA damage from increased reactive oxygen species) in rats that underwent the surgery. Additionally, they also found that the proportion of neurons expressing 8oxoG DNA was significantly decreased in the rats treated with riluzole. These findings support the theory that ROS play a key role in the development of WCS [11]. 

We know for a fact that our patient’s type of compression was of a chronic and prolonged nature (i.e. OPLL), which could have modified the surrounding vasculature, causing chronic local ischemia. In a systematic review done by Bagherzadeh et al, they found that some risk factors for WCS were advanced age, extensive surgery, posterior approach for decompression, and the presence of OPLL, all of which were present in our patient [12]. There are some patterns reported in the literature about WCS, such as preoperative hyperintense changes in T2-weighted MRI at the level of the lesion and chronic compression. With an increased accessibility to imaging modalities such as high-resolution MRI, we could pay special attention to patients presenting with T2-weighted hyperintensity in preoperative imaging. 

Unfortunately, our patient died due to complications associated with quadriplegia and cervical cord injury: poor airway secretion management, formation of mucus plug, pneumonia with subsequent severe sepsis, and loss of vascular tone causing profound hypotension, and thus, hypoxia. Unlike the previous cases reported in the literature, our patient did not have changes in preoperative spinal cord T2-weighted MRI. During surgery, neuromonitoring signals were lost. After surgery, he developed extensive spinal cord edema after decompression, as seen in the postoperative MRI. This led to profound quadriparesis, which compromised the autonomic nervous system, resulting in impaired airway secretion management and hemodynamic instability. Despite starting IV steroid treatment early and increasing MAP with vasopressors, our patient did not improve and ultimately passed away. If no iatrogenic injury can be associated with a sudden loss of neuromonitoring signals intraoperatively (e.g. bone fragment invading the spinal canal, dural tear, epidural hematoma, misplaced pedicle screw, etc.), surgeons must suspect WCS in patients with high risk factors. Early suspicion will lead surgeons to treat the signs and symptoms early in their progression. If neuromonitoring signals are lost, increasing MAP above 85mmHg and administration of intravenous steroids have been proved to be beneficial. Additionally, initiating physical therapy in the subacute period is of utmost importance for the adequate recovery of patients suffering from any spinal cord injury.

## Conclusions

WCS is a rare but serious complication of decompression surgery. Our patient presented with profound weakness of the upper and lower extremities and RUE plegia immediately after surgery, as previously described in the postoperative physical examination. It is important to note that while preoperative T2 hyperintensities on MRI are a common finding in the reported cases of WCS, its absence does not exclude the risk of developing it (as seen in our patient). Our patient suffered from a chronic pathology (i.e., OPLL), advanced age, and preoperative weakness, all of which have been reported as possible risk factors. Another risk factor may be T2-weighted hyperintensity changes, which were not seen in our case. Surgeons assessing patients for these risk factors may prepare for the potential development of WCS and act accordingly. Potential therapeutic strategies such as maintaining mean arterial pressure, administering high-dose steroids, and placing a CSF shunt have been shown to be effective in some cases, reducing cord edema and ischemia. However, there is no consensus on the management of WCS. Future research, including biochemical and case-control studies, is required to gain more insight into the processes of WCS and establish effective prevention strategies. This report aims to raise awareness among spine surgeons about this rare but critical complication in an effort to improve patient outcomes.
